# Energy expenditure differences across lying, sitting, and standing positions in young healthy adults

**DOI:** 10.1371/journal.pone.0217029

**Published:** 2019-06-12

**Authors:** Francisco J. Amaro-Gahete, Guillermo Sanchez-Delgado, Juan M. A. Alcantara, Borja Martinez-Tellez, Francisco M. Acosta, Elisa Merchan-Ramirez, Marie Löf, Idoia Labayen, Jonatan R. Ruiz

**Affiliations:** 1 EFFECTS-262 Research Group, Department of Medical Physiology, School of Medicine, University of Granada, Granada, Spain; 2 PROmoting FITness and Health through physical activity research group (PROFITH), Department of Physical Education and Sports, Faculty of Sport Sciences, University of Granada, Granada, Spain; 3 Division of Endocrinology, and Einthoven Laboratory for Experimental Vascular Medicine, Department of Medicine, Leiden University Medical Center, Leiden, The Netherlands; 4 Department of Biosciences and Nutrition, Karolinska Institutet, Huddinge, Sweden; 5 Department of Health Sciences, Public University of Navarra, Pamplona, Spain; University of Pavia, ITALY

## Abstract

The time spent in sedentary behaviour represents an important public health burden. To reduce sedentary time in the general population, the simplest, most effective, and most accessible method is to decrease lying and sitting time. We aimed to compare differences on energy expenditure (EE) across lying, sitting, and standing positions; and to analyse the associations between the change on EE of changing from one position to another and anthropometric and body composition parameters in young healthy adults. A total of 55 (69% women) young healthy adults aged 21.7 ± 2.2 participated in the study. We measured EE by indirect calorimetry across lying, sitting, and standing positions following the standard procedures. The EE was significantly higher in standing than in both lying and sitting positions (mean difference: 0.121±0.292 and 0.125±0.241 kcal/min, respectively; all P<0.001), and no differences were observed between lying and sitting positions (P = 1.000). There was a negative association between the EE differences in sitting vs. standing position and lean body mass (P = 0.048), yet no associations between EE differences with the rest of the anthropometric and body composition parameters were observed in each position pair studied (all P>0.321). Our findings support the fact that increasing the time spent standing could be a simple strategy to slightly increase EE. Therefore, our results have important clinical implications including a better monitoring, characterizing, and promoting countermeasures to sedentariness through low-level physical activities.

## Introduction

The time spent in sedentary behaviour is increasing in modern societies, and it currently represents an important public health burden [1]. Sedentary lifestyle is a risk factor for all-cause mortality [2–4], metabolic syndrome or type 2 diabetes [5], obesity [6], and even cancer [7], independently of physical activity levels [2]. To reduce sedentary time in the general population, the simplest, most effective, and most accessible method is to decrease lying and sitting time [8].

Although it is commonly believed that lying, sitting, and standing require a different energy expenditure (EE), there is still controversy. Whereas some studies reported no EE differences between lying and sitting [9,10], others have shown that EE was 20% higher in lying than in sitting [11]. On the other hand, replacing sitting with standing is recommended to decrease sedentary time and increase the daily energy expenditure [12,13], but the difference in EE between sitting and standing also remains controversial. When comparing sitting vs. standing, studies show EE differences ranging from 10 to 100% [14–16]. These contradictory findings might be partially explained by the lack of a rigorous control in the experimental design, data collection (i.e. different gas collection system), and/or data analysis [8,16,17]. Other variables, such as anthropometric characteristics, body composition, age, or sex, may have also contributed to these discrepancies, but their role is largely unknown. There is a lack of studies showing the EE differences in the three positions, lying, sitting, and standing, and it would be of public health interest to better understand the EE changes across positions.

The objectives of the present study were (i) to compare differences on EE across three positions: sitting, lying, and standing, and (ii) to determine the associations between the change on EE across these postures (lying vs. Sitting, lying vs. Standing, and sitting vs. Standing) with anthropometric and body composition parameters in young healthy adults.

## Material and methods

### Study participants

The participants were enrolled in the ACTIBATE study [18] (ClinicalTrials.gov ID: NCT02365129) and met the following inclusion criteria: (i) being non-smokers, (ii) not taking any medication, (iii) not having an acute or chronic illness, and (iv) not being pregnant. The study was approved by the Human Research Ethics Committee of both the University of Granada (n°924) and Servicio Andaluz de Salud (Centro de Granada, CEI-Granada), and complied with the revised ethical guidelines of the Declaration of Helsinki (revision of 2013). All participants signed the written informed consent before their enrolment. A total of 84 young healthy sedentary adults aged between 18 to 25 years old were recruited for the current study. We had some technical problems in data collection with specific participants (i.e. calibration of the gas analyzer), and we excluded some participants because they did not strictly meet the standardized previous conditions (i.e. fasting time, physical activity, drugs or supplements intake etc) or because they talking or moving during the data collection. Thirty-one individuals were excluded and, therefore a total of 53 participants were included in the analysis.

### Procedures

The study was conducted between April and June 2017. The participants were instructed to refrain from any moderate or vigorous physical activity within 24 and 48 hours, respectively, before the testing day, and not to consume caffeine and/or dietary supplements in the 24 hours prior to testing. The participants arrived to the laboratory by car or by bus (avoiding any physical activity after waking up) and in a fasted state (between 5 and 6 hours).

**Fig 1:**
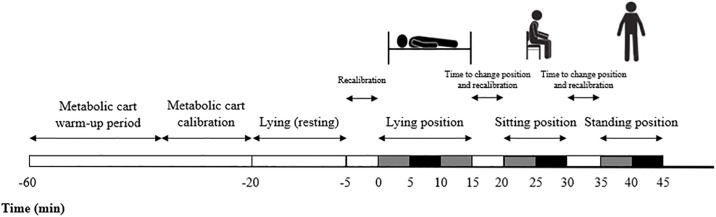
Experimental study design. White areas represent the time periods when energy expenditure was not registered; grey areas included the time periods when the energy expenditure measurement was performed but discarded; black areas represent the time periods when energy expenditure measurement was performed and selected for further analysis.

The experimental design can be seen in Fig 1. The EE measurements were performed by indirect calorimetry following the current recommendations [19]. Briefly, all the measurements were carried out in the same room and by the same trained staff. Before being evaluated, all the participants confirmed that they had met the previous study conditions. Then, they lay on a reclined stretcher in a supine position during the 5–10 previous minutes to the indirect calorimetry measurement. They were instructed to breathe normally, and not to talk, fidget, or sleep. The same position and instructions were maintained during the next 15 minutes, when the indirect calorimetry measurements were performed. Afterwards, the participants sat on a supported chair with flat back placed near the bed during 10 minutes following the same instructions mentioned previously. Finally, the participants were asked to stand-up slowly and avoid unnecessary movements. In this static position, indirect calorimetry was measured for another 10 minutes. The transitions from lying to sitting, and from sitting to standing took a maximum of 5 minutes which were removed from the analysis.

Indirect calorimetry measurements were performed with the CPX Ultima CardiO2 (Medical Graphics Corp, St Paul, USA), using an oronasal mask (model 7400, Hans Rudolph Inc, Kansas City, MO, USA), equipped with a prevent metabolic flow sensor (Medgraphics Corp, Minnesota, USA) [20,21]. Flow calibration was performed using a 3-L calibration syringe at the beginning of every testing day, and the gas analyser was calibrated using two standard gas concentrations following the manufacturer’s instructions before each EE measurement i.e. lying, sitting, and standing. The data obtained from the indirect calorimetry assessment included: EE (calculated by the Weir abbreviated equation [assuming negligible protein oxidation] and expressed as Kcal/day [22]: EE = [3.9 (VO2) + 1.1 (VCO2)] * 1.44), respiratory quotient (RQ), minute ventilation (VE), and respiratory rate (RR). Heart rate (HR) was recorded with a heart-rate monitor (Polar RS800CX, Polar Electro, Kempele, Finland) in all measurements.

The weight (±10 g) and height (±5 mm) were measured without shoes and with light clothing, using a digital integrating scale (SECA 760, Hamburg, Germany), and a stadiometer (SECA 220, Hamburg, Germany). Body mass index (BMI) was calculated as weight (kg)/height (m^2^), and body composition (lean body mass and fat body mass) was determined by Dual Energy X-ray Absorptiometry (HOLOGIC, Discovery Wi). A detailed explanation of body composition procedures can be found elsewhere [23–25]. Lean and fat body mass percentage was calculated as lean body mass (kg)*100/weight (kg), and fat body mass (kg)*100/weight (kg), respectively.

### Data analysis

The gas exchange parameters were averaged every minute with the Breeze Suite (version 8.1.0.54 SP7, MGC Diagnostic) software. Later, we discarded the first 5 minutes record in each position and averaged the obtained data from the 6^th^ to the 10^th^ minute, which has been previously showed to be a valid option for indirect calorimetry data analysis [26].

To determine the interindividual variability in response to the change on EE of changing from one position to another (lying vs. sitting, lying vs. standing, and sitting vs. standing), the participants were also categorised as spenders and savers [8,27]. ‘Spender’ refers to a participant with a rise in EE >5% between two positions which is maintained during the entire assessment period, and ‘saver’ refers to those who showed little or no change in EE (a rise in EE <5%) between two positions [8,27].

### Statistical analysis

We performed a sample size calculation based on a minimum predicted change of 5% in EE between lying and standing positions, and an SD for this change of 10%. A sample size of 40 participants was predicted to provide a statistical power of 80% considering a type I error of 0.05 [28]. Therefore, a sample size of 53 participants was enough to test our hypothesis. We conducted repeated measures analyses of variance (ANOVA) to compare EE, RQ, VE, RR, and HR across positions (lying, sitting, and standing), using Bonferroni post-hoc comparisons. The differences between variables were computed in all cases as: (i) standing—lying, (ii) standing—sitting, and (iii) sitting—lying. Linear regressions were conducted to examine if the EE differences between two positions could be explained by anthropometric or body composition parameters. We also conducted t-student unpaired-samples test to study the differences between spenders and savers.

The analyses were conducted using the Statistical Package for Social Sciences (SPSS, v. 21.0, IBM SPSS Statistics, IBM Corporation), and the level of significance was set at P<0.05.

## Results

Table 1 shows the descriptive characteristics of the study participants. No interaction by sex was observed in EE, RQ, VE, RR, and HR changes across all positions studied (all P interaction≥0.477).

**Table 1:** Descriptive parameters.

	All (n = 53)	Men (n = 15)	Women (n = 38)
Age (years)	21.8 ± 2.3	22.2 ± 2.6	21.5 ± 2.1
Body mass (kg)	71.8 ± 15.3	82.7 ± 15.5	67.5 ± 12.6*
Height (cm)	168.7 ± 7.9	177.1 ± 6.3	165.4 ± 5.7*
BMI (kg/m^2^)	25.2 ± 4.8	26.4 ± 11.2	24.7 ± 4.5
Lying EE (kcal/min)	1.20 ± 0.24	1.44 ± 0.26	1.10 ± 0.15*
Sitting EE (kcal/min)	1.19 ± 0.22	1.40 ± 0.24	1.11 ± 0.15*
Standing EE (kcal/min)	1.32 ± 0.29*	1.59 ± 0.31	1.21 ± 0.21*
Lean body mass (kg)	41.68 ± 8.41	51.98 ± 6.20	37.61 ± 4.99*
Fat body mass (kg)	26.29 ± 9.09	26.07 ± 11.18	26.37 ± 8.30
Fat body mass (%)	36.79 ± 7.84	30.96 ± 8.92	39.08 ± 6.09*

Data are expressed as mean ± standard deviation. Abbreviations: BMI, body mass index; EE; energy expenditure.

*P<0.05 (analysis between sex by t-Student unpaired-samples test).

**Fig 2:**
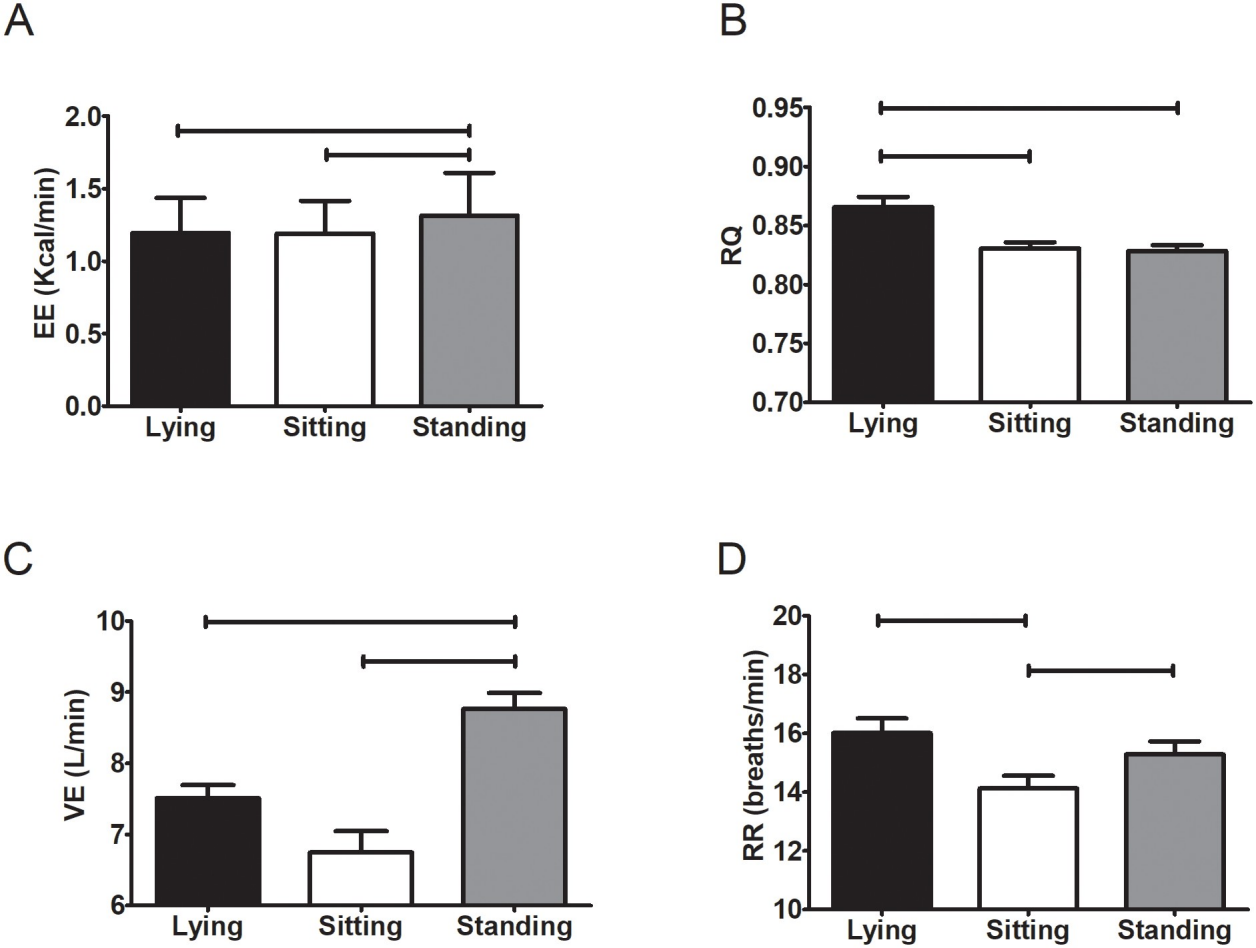
Energy expenditure (EE; panel A), respiratory quotient (RQ; panel B), minute ventilation (VE; panel C), and respiratory rate (RR; panel D) in lying, sitting, and standing positions. Values are expressed as mean ± standard deviation. P value derived of repeated measures analysis of variance followed by the Bonferroni post hoc test. Parallel lines mean P<0.001.

**Fig 3:**
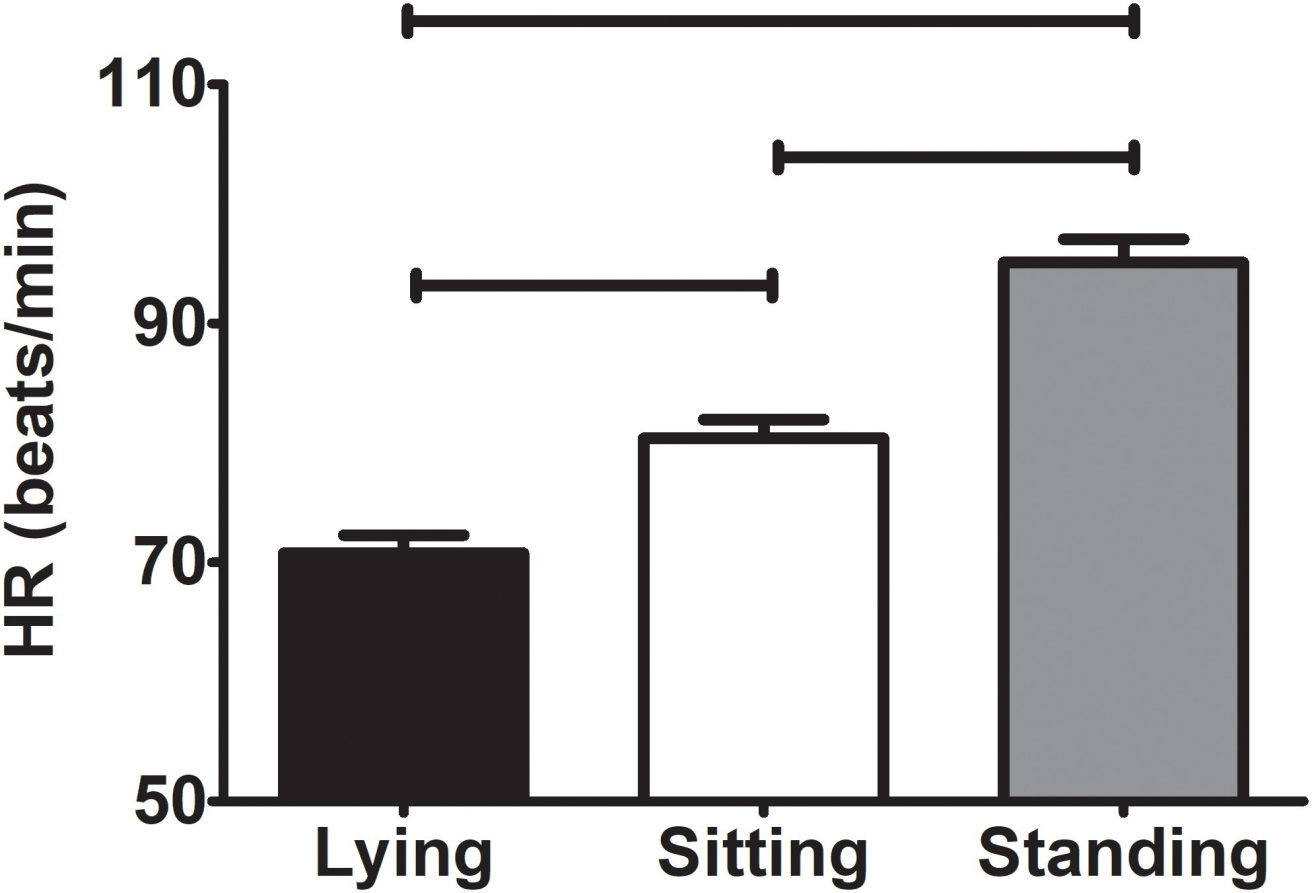
Heart rate (HR) in lying, sitting, and standing positions. Values are expressed as mean ± standard deviation. P value derived of repeated measures analysis of variance. Parallel lines mean P<0.001.

Fig 2 shows the mean values of EE, RQ, VE, and RR in lying, sitting, and standing positions. The EE was significantly higher standing than in lying and sitting positions (mean difference: 0.121±0.292 and 0.125±0.241 kcal/min, respectively; change percentage: 9.7±10.9 and 10.2±11.6%, respectively; all P<0.001), and no differences were observed between lying and sitting positions (P = 1.000). The RQ was higher lying than in sitting and standing positions (mean difference: 0.04±0.06 and 0.04±0.05, respectively; change percentage: 3.7±6.8 and 4.0±6.0%, respectively; all P<0.001). The VE was higher standing than in lying and sitting positions (mean difference: 1.22±0.88 and 1.27±0.94 L/min, respectively; change percentage: 13.5±9.2 and 13.9±9.7%, respectively; all P<0.001), yet no differences were found between lying and sitting positions. Furthermore, there were differences in RR between lying and sitting positions, and between sitting and standing positions (mean difference: 1.88±2.65 and 1.18±2.02 breath/min, respectively; change percentage: -14.93±20.65 and 6.90±13.77%, respectively; all P<0.001); yet no differences were observed between lying and standing positions (P = 0.295). Moreover, The HR was significantly higher standing than in sitting (mean difference: 16±8 beats per minute; change percentage: 16.6±9.1%; P<0.001) and lying positions (mean difference: 25±15 beats per minute; change percentage: 26.0±17.8; P<0.001). The HR was also higher sitting than lying (mean difference: 9.2±6.4 beats per minute; change percentage: 11.3±8.7; P<0.001) (see Fig 3).

**Fig 4:**
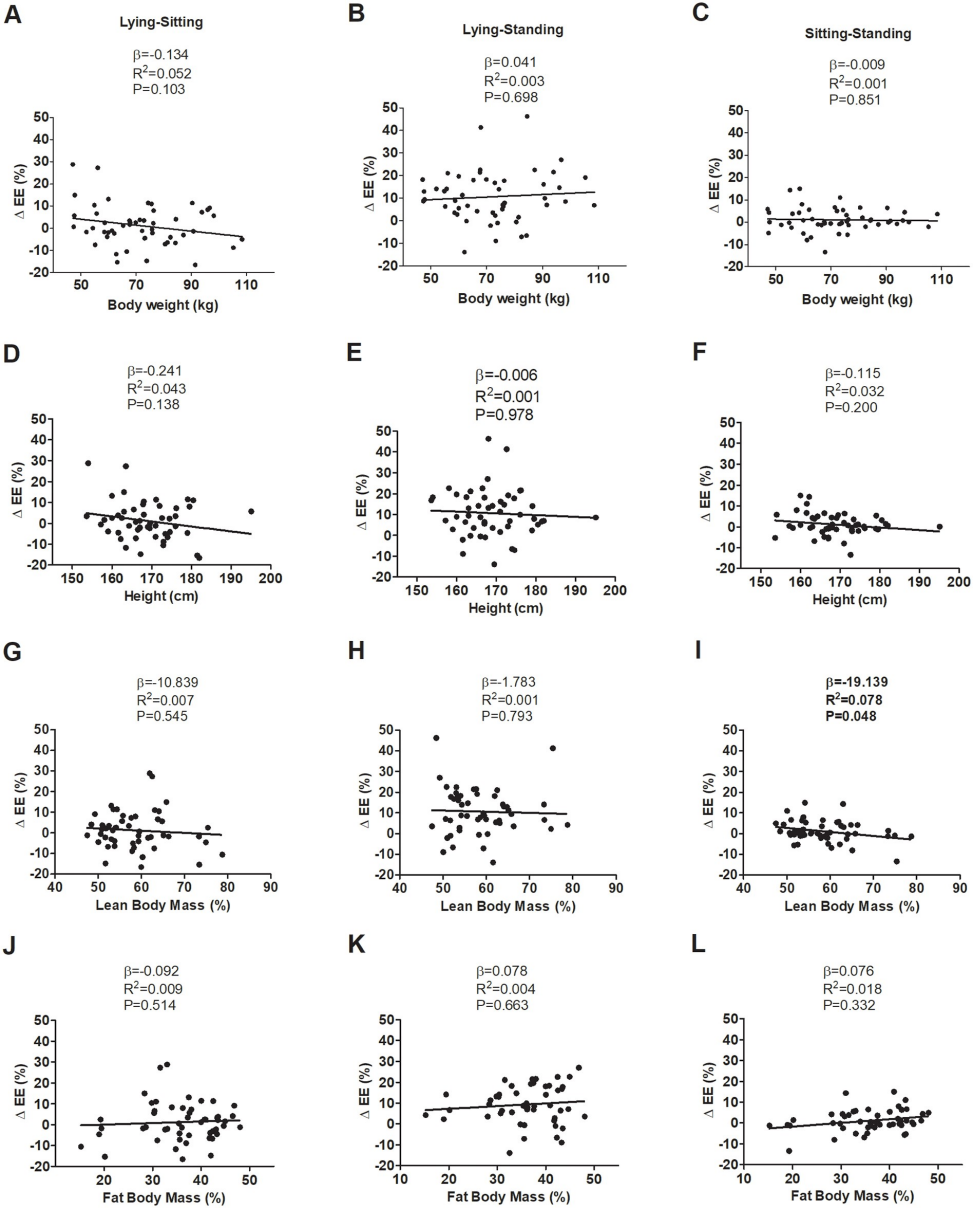
Associations between the change on energy expenditure (EE) of changing from one position another: Lying vs. sitting, lying vs. standing, and sitting vs. standing with body weight (Panels A, B, and C), height (Panels, D, E, and F), lean body mass (Panels G, H and I), and fat body mass (Panels J, K and L).

Fig 4 shows the association between the change on EE of changing from one position to another in all different position pairs (i.e. lying vs. sitting; sitting vs. standing; sitting vs. standing) and the anthropometric and body composition parameters. There was a significant negative association between the EE differences in sitting vs. standing position and lean body mass (Fig 4I) (R^2^ = 0.078, P = 0.048), yet no significant associations between EE differences with the rest of the anthropometric and body composition parameters were observed in each position pair studied (all P>0.321). We also noticed no significant associations between the RQ differences in all different position pairs and the anthropometric and body composition parameters (see S1 Fig) (all P>0.321).

**Fig 5:**
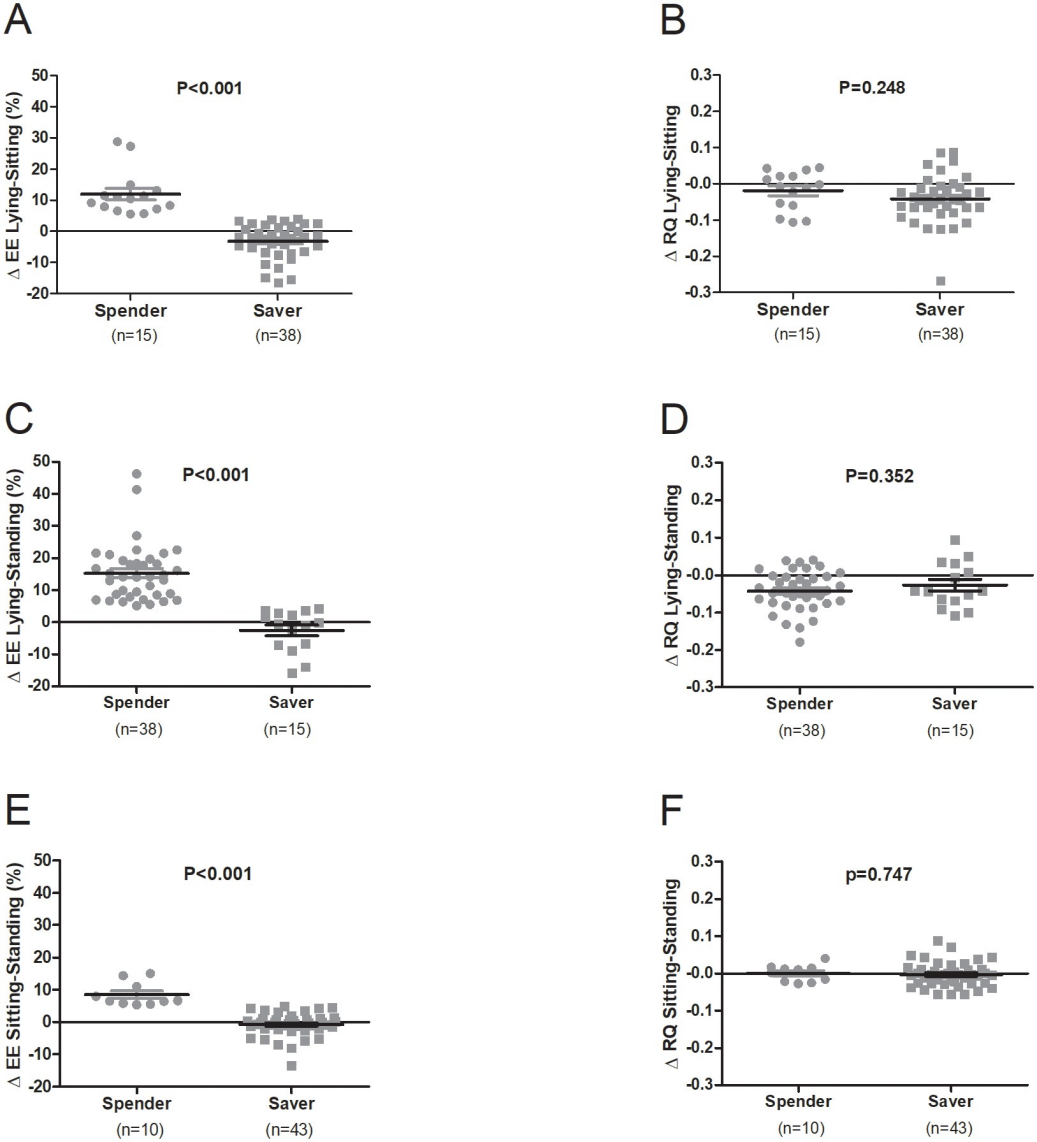
R individual energy expenditure (EE; Panels A, C, and E) and respiratory quotient (RQ; Panels B, D, and F) responses of lying vs. sitting position (Panels A and B), lying vs. standing position (Panels C and D) and sitting vs. standing position (Panels E and F) comparing spenders vs. savers. Spenders were defined as participants who showed a rise in EE >5% between two positions and savers are those who showed little or no change in EE (a rise in EE of <5% between two positions). Δ EE was calculated as the change in EE during the second 5 minutes corresponding to the two positions compared, and it is expressed as a percentage of the first position indicated for each comparison. Δ RQ was calculated as the change in RQ during the second 5 minutes corresponding to the two positions compared, and it is expressed as means ± standard deviation.

According to the EE change from lying to sitting, 71.7% of participants (n = 38) were classified as savers, and only 28.3% (n = 15) were spenders (EE change in savers and spenders: -3.2±5.5% vs. 11.9±7.1%, respectively; P<0.001; Fig 5A). By definition, the EE change from lying to standing was higher in the spenders group (spenders: 71.7%, n = 38; mean change -2.6±6.5% and 15.3±9.0%, savers and spenders, respectively, P<0.001; Fig 5C). Furthermore, the EE change from sitting to standing was also higher in the spenders group (spenders: 18.9%, n = 10; -0.8±3.6; vs. 8.5±3.7%, savers and spenders, respectively, P<0.001; Fig 5E). There were no significant differences between savers and spenders in RQ in any of the three pairs of positions (all P>0.248) (see Fig 5B, 5D and 5F).

We also studied the anthropometric and body composition parameters as well as the EE lying, sitting, and standing in savers and spenders, and in the three pairs of positions studied (see Table 2): (i) lying vs. sitting, (ii) lying vs. standing, and (iii) sitting vs. standing. There were no significant differences in age, BMI, lean body mass and fat body mass, except when comparing saver vs. spender in sitting vs. standing position in term of lean body mass (42.65±8.60 vs. 37.50±6.32 kg respectively, P<0.05). We found significant differences in EE between all of the three position pairs established between saver and spender. All of these findings persisted after controlling for the time of the day when the test was performed and for the menstrual cycle period in women (data not shown). Interestingly, we also found that EE relative to lean body mass was higher in women compared with men in lying, sitting and standing position (0.032±0.008, 0.032±0.007, 0.035±0.009 vs. 0.025±0.006, 0.024±0.00, 0.026±0.006, respectively, all P>0.01).

**Table 2:** Demographic characteristics in savers and spenders.

	All (n = 53)	SAV lying-sitting (n = 38)	SPE lying-sitting (n = 15)	SAV lying-standing (n = 15)	SPE lying-standing (n = 38)	SAV sitting-standing (n = 43)	SPE sitting-standing (n = 10)
Age (years)	21.7 ± 2.2	21.8 ± 2.2	21.5 ± 2.4	21.1 ± 2.2	21.9 ± 2.2	21.9 ± 2.2	21.1 ± 2.4
Sex (%)							
Men	15 (28.3)	10 (66.6)	5 (33.3)	3 (20)	12 (80)	15 (100)	0 (0)
Women	38 (71.7)	28 (73.7)	10 (26.3)	12 (31.6)	26 (68.4)	28 (73.7)	10 (26.3)
Weight (kg)	71.8 ± 15.3	72.0 ± 13.5	71.3 ± 19.7	71.8 ± 8.4	71.8 ± 17.4	72.9 ± 15.8	67.2 ± 13.0
Height (cm)	168.7 ± 7.9	168.0 ± 6.7	170.5 ± 10.2	167.5 ± 6.0	169.2 ± 8.5	169.7 ± 7.7	164.4 ± 7.2
BMI (kg/m^2^)	25.2 ± 4.8	25.5 ± 4.5	24.2 ± 5.3	25.6 ± 2.9	24.9 ± 5.3	25.2 ± 4.9	24.7 ± 3.8
Lean body mass (kg)	41.68 ± 8.41	41.80 ± 8.20	41.36 ± 9.21	41.69 ± 6.70	41.67 ± 9.08	42.65 ± 8.60^a^	37.50 ± 6.32^a^
Fat body mass (kg)	26.29 ± 9.09	26.49 ± 8.75	25.79 ± 10.22	26.31 ± 8.01	26.29 ± 9.59	26.32 ± 9.56	26.16 ± 7.16
Fat body mass (%)	36.79 ± 7.84	37.04 ± 8.66	36.14 ± 5.42	37.16 ± 9.53	36.64 ± 7.20	36.20 ± 8.30	39.30 ± 4.96

Data are expressed as mean ± standard deviation. Abbreviations: SAV, savers (a rise in energy expenditure of <5% between two positions compared); SPE, spenders (a rise in energy expenditure of >5% between two positions compared); BMI, body mass index. Values sharing superscript (i.e. “a”) are significantly different (p<0.05) from another by t-Student unpaired-samples test.

## Discussion

The main findings of this study showed that standing increases EE above sitting and lying values (~10%), while sitting and lying paradoxically seems to represent similar EE. Taken together, these findings suggest that decreasing lying and sitting times could be a simple strategy to slightly increase energy expenditure.

Our results concur with those of others who also showed higher EE when standing than when sitting [8]. However, Monnard et al. [17] reported that a multi-ethnic male cohort had the same EE when comparing the sitting and standing positions. Our results also indicate that EE is similar in sitting compared to lying in young healthy adults, which concurs with other studies [9,10,29]. Similarly, although no differences were found in EE between lying and sitting, we found lower RQ values when the participants were sitting compared with when they were lying. The changes (decrease) in RQ from lying to sitting, and from lying to standing had been previously found [8,10], but it remains necessary to thoroughly investigate the physiological mechanisms that could explain such changes. Our results suggest that the lack of differences in EE between lying and sitting, and higher values in RQ in the former could be due to an extra activation of ventilatory muscles (e.g. diaphragm) and to a loss of ventilatory efficiency in lying vs. sitting [30], which would also be reflected in higher RR in lying position. Postural maintenance needs a higher muscle activation sitting than lying. Therefore, a higher EE would be expected when sitting than when lying. We hypothesise that gravity-induced displacement of viscera weight against the diaphragm would cause and over-load to ventilatory muscles which ultimately contributes to a higher EE, compensating the relaxed postural muscles [31]. This could be reflected in higher RR while lying, despite similar VE (i.e. decrease in ventilatory efficiency). When the ventilatory muscles are slightly over-loaded, they would need to recruit fast fibers (which involve carbohydrate oxidation metabolism) in higher proportion than postural muscles, which would explain why we found higher RQ but similar energy expenditure in lying than in sitting positions. However, it is well-known that RQ is heavily influenced by VE and that hyperventilation leads to more clearance of CO2 obtaining a higher RQ. This issue is a reflection of breathing pattern but not a change in substrate utilization. Therefore, these findings should be deeply investigated in future studies.

Paradoxically, our results presented a large inter-individual variability showing lower EE during standing compared to lying in a number of participants (n = 7). A possible explanation for this finding could be that the initial lying assessment had not reached a steady-state, thereby overestimating the measurement [8]. We showed that a total of 14 participants did not meet the steady state criteria [19], but after sensivity analysis excluding those individuals that did not attain the steady state, the results persisted. Further studies are needed to deeply investigate this concern.

Interestingly, most participants in our study showed a small or even no rise in the EE when standing compared to sitting, being categorised as savers a total of 81% of the study participants, which concurs with other studies [17]. The mechanism by which the large majority of participants appear to be spenders remains to be elucidated, but anthropometric and body composition variables may, in part, be related to these phenotypes.

We did not find any association between EE differences and almost every anthropometric, or body composition variables when compared lying vs. sitting, and lying vs. standing position. However, despite the inter-individual variability among participants in terms of EE, this study indicates that when compared sitting vs. standing position, EE differences could be explained in part by lean body mass. Miles-Chan et al. [8] compared energy cost in standing vs. sitting positions, and no associations were found between the EE differences and anthropometry (body weight or height) in 22 young adults with normal BMI, which concurs with our findings. However, a recent study [17] showed that the body weight and the leg length might contribute to the inter-individual variability in the EE (R^2^ = 0.548; P = 0.001, and R^2^ = 0.460; P = 0.006, respectively) considering sitting vs. standing positions, but it was not taken into account body composition parameters in the regression analysis. We found a significant negative correlation between the EE differences in sitting vs. standing position and lean body mass. Our results could be explained by the fact that lean body mass is positively correlated with efficiency in term of EE [32]. Therefore, the present study supports the argument that individuals with lower lean body mass have lower EE in resting condition (i.e. lying position), but show higher EE differences considering sitting vs. standing position.

There are clinical and research implications derived from these findings. Firstly, in order to develop several strategies related to fighting against metabolic diseases related to energy balance, it is important to consider that the EE is higher when standing than when lying and sitting. Secondly, the EE can be accurately determined in both lying and sitting positions, but it is partially incorrect when the aim is to describe the nutrient oxidation rate, because RQ was not comparable between positions. Therefore, it is tempting to speculate that lying with a specific bed inclination could be the best approach to determine the “real” EE, since this position would help to avoid extra activation of ventilatory muscles increasing ventilatory efficiency, and also contributing to a minimum recruitment of postural muscles [31].

The results of this study should be considered with caution as there are some limitations. Although we standardised the protocol test, the order of the positions was not randomised and a drag effect may have been produced. We carefully controlled the fasting time (5–6 hours) prior to the test, but the best practice guidelines suggest to established at least 7 hours. Moreover, the composition of the previous meal was not standardised, and this fact may have influenced the RQ measurement [33]. The shorter supine resting period prior to the test protocol may have affected subsequent measurements during the lying position and thus the lying vs. sitting comparison. In addition, although the majority of the participants of the current study met the steady state criteria in lying (73.6%), sitting (58.5%), and standing (62.3%), there was a number of individuals that did not meet the above-mentioned criteria causing a potential overestimation of EE in all postures.

In conclusion, our findings support that increasing the time spent standing could be a simple strategy to increase the EE. In fact, it is clear that reducing sitting time should be encouraged according to estimations indicating that substituting 6 hours of sitting per day with standing results in 45 additional kcal in daily energy expenditure. Therefore, our findings have important clinical implications including a better monitoring, characterizing, and promoting countermeasures to sedentariness through low-level physical activities.

## Supporting information

S1 Fig Relationship between the change on respiratory quotient (RQ) of changing from one position to another: Lying vs. sitting, lying vs. standing, and sitting vs. standing with body weight (Panels A, B, and C), height (Panels D, E, and F), lean body mass (Panels G, H, and I), and fat mass (Panels J, K, and L). (TIFF)

S1 File Study´s data base. (SAV)
