# Correction: Energy expenditure differences across lying, sitting, and standing positions in young healthy adults

**DOI:** 10.1371/journal.pone.0219372

**Published:** 2019-07-01

**Authors:** Francisco J. Amaro-Gahete, Guillermo Sanchez-Delgado, Juan M. A. Alcantara, Borja Martinez-Tellez, Francisco M. Acosta, Elisa Merchan-Ramirez, Marie Löf, Idoia Labayen, Jonatan R. Ruiz

The last author's name is indexed incorrectly in PubMed. The correct name is: Ruiz JR.
