# Supplementary figures and images for: Energy expenditure differences across lying, sitting, and standing positions in young healthy adults
Source: PLoS One. 2019 Jun 12;14(6):e0217029. doi: 10.1371/journal.pone.0217029 (PMC6561541; doi:10.1371/journal.pone.0217029)

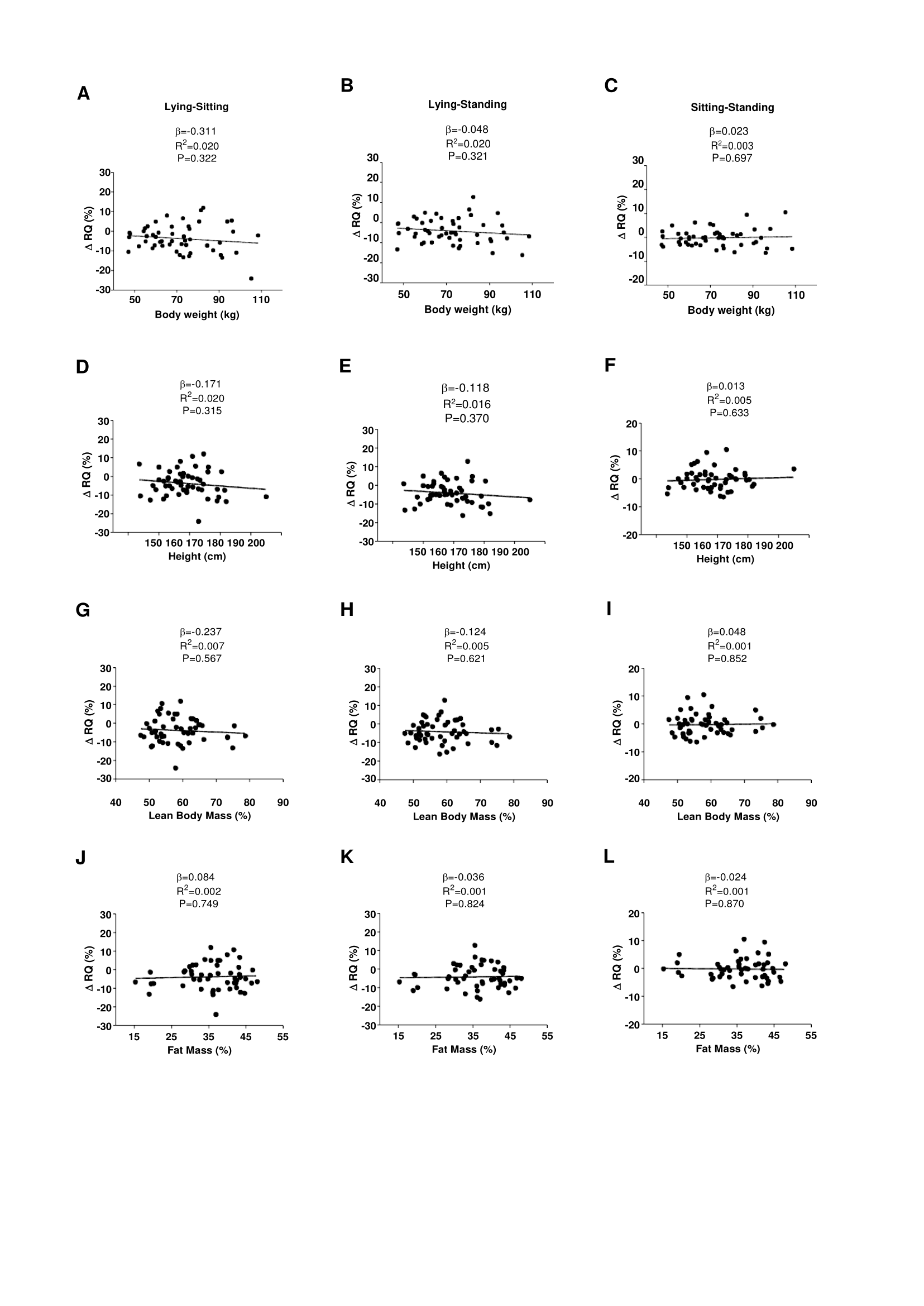

Supplement: S1 Fig — (TIFF) [file pone.0217029.s001.tiff]
